# Perceptions on support, challenges and needs among parents and caregivers of children with developmental disabilities in Croatia, North Macedonia and Serbia: a cross-sectional study

**DOI:** 10.1186/s12887-024-04770-7

**Published:** 2024-05-03

**Authors:** Jelena Međaković, Antonia Čivljak, Tatjana Zorčec, Vesna Vučić, Danijela Ristić-Medić, Aleksandra Veselinović, Marta Čivljak, Livia Puljak

**Affiliations:** 1 Centre for Evidence-Based Medicine and Health Care, Catholic University of Croatia, Ilica 242, Zagreb, 10000 Croatia; 2 Institute of Emergency Medicine of Zagreb County, Velika Gorica, Zagreb, Croatia; 3 University Children’s Hospital Medical Faculty, Skopje, North Macedonia; 4 Centre of Research Excellence in Nutrition and Metabolism, Group for Nutritional Biochemistry and Dietology, Institute for Medical Research, National Institute of the Republic of Serbia, University of Belgrade, Belgrade, Serbia; 5 Centre of Research Excellence in Nutrition and Metabolism, Group for Nutritional Biochemistry and Dietology, Institute for Medical Research, National institute of Republic of Serbia, University of Belgrade, Belgrade, Serbia; 6 Cognitive Neuroscience Department, Research and Development Institute “Life Activities Advancement Centre”, Belgrade, 11000 Serbia

**Keywords:** Caregivers, Children with developmental disabilities, Perception, Challenges and needs

## Abstract

**Background:**

Parents/caregivers of children with developmental disabilities (CDD) have a wide range of support needs and there are various interventions available. Support, challenges, and needs among parents/caregivers of CDD likely vary in different geographical settings. This study aimed to analyze the perceptions of support, challenges, and needs among parents/caregivers of CDD in Croatia, North Macedonia, and Serbia.

**Methods:**

We conducted a cross-sectional study in March-April 2023 within the Erasmus + SynergyEd project. The eligible participants were parents and caregivers of CDD in Croatia, North Macedonia, and Serbia, who filled out a modified Caregiver Needs Survey online.

**Results:**

Among 953 participants, 542 (57%) were from Croatia, 205 (21%) were from North Macedonia and 206 (22%) were from Serbia. The most common diagnosis of participants’ children was autism spectrum disorder (26%). The child most often received the first diagnosis at the median of 2 years, diagnosed by a team of professionals. More than half (58%) of children attended preschool and public school, while 22% did not attend any schooling. Additional support from the state/city/county was received by 66% of CDD. Most participants declared not participating in association/organization for family support. Participants mostly (68%) used experts who work with the child as a source of information about their child’s condition, followed by the Internet (53%). In the last 12 months, 60% of participants had difficulties with the availability of services in their area or problems getting appointments. The biggest problem in getting support was ensuring the child’s basic rights were protected. Participants stated that ensuring greater rights for CDD was the greatest need for their families.

**Conclusion:**

Parents/caregivers of CDD in Croatia, North Macedonia, and Serbia faced multiple challenges, but most of them were satisfied with the services provided to their children. Future efforts to develop policies and services related to CDD should consider the opinions of their parents/caregivers and disparities in access to services.

**Supplementary Information:**

The online version contains supplementary material available at 10.1186/s12887-024-04770-7.

## Background

Children with developmental disabilities (CDD) are a growing group of children who have or are at increased risk of chronic physical, developmental, behavioral, or emotional conditions, requiring healthcare and related services of a type or amount beyond that required by children generally [1].

Parents of CDD face numerous difficulties in their daily lives. They experience stress while managing their children’s behaviors and needs, and their family’s dynamic changes due to dealing with their children’s various requirements [2, 3]. Stress that is carried over a long period affects every element of life and may even cause non-functional reactions in the individual [4]. They also feel social isolation and misunderstanding of the family situation by other people, and difficulties in talking to others, including physicians and nurses [5]. A report published in 2022, which analyzed data from the US 2016–2018 National Survey of Children’s Health Dataset showed that mothers of children with special needs had worse health compared to mothers with healthy children [6].

Parents and caregivers of CDD play crucial roles in their children’s lives, especially in a child’s learning, self-esteem, and attitude toward lifelong learning [7, 8]. Caregiver’s support is positively correlated with children’s academic, learning, social, and behavioral outcomes [9, 10].

Caregivers of CDD experience challenges that may be exacerbated by challenging behavior (CB) and insufficient access to specialized support services. While waiting for services, caregivers must implement strategies on their own, while taking arduous steps to access supports to eventually meet their needs [11].

Caregivers of CDD have a wide range of support needs. They have substantial needs for services whose aim is to bolster their capacity to provide support, and also to achieve a high quality of life. Thus, there is a compelling need for evidence-based practice to provide such support [12].

In 2021, Papoudi et al. published a scoping review about perceptions, experiences, and needs of culturally and linguistically diverse families of children with autism [13]. They found that social stigma and barriers to treatment access are two factors that impact families’ perceptions of autism and are typically attributed to a lack of information. The creation of culturally aware interventions, multilingual information, and parent-professional collaboration were all indicated as needs. These results have implications for practice, policy, and research [13].

In 2020, Elangkovan and Shorey published a systematic review of the experiences and needs of parents of children with cerebral palsy [14]. Four main themes emerged, related to self-care (accepting the situation, dealing with the physical and emotional demands, finding a good outlet), family (balancing raising several children and raising oneself as a full-time job), society (public discrimination, rejection by extended family, and loss of independence) and parents’ wish list regarding the open communication and compassionate attitudes, funding and health care accessibility, social integration, and inclusivity. Parents expressed a need for more money, health care services information, better attitudes and empathy from healthcare providers, appropriately qualified educators, disability-friendly fixtures, and increased public knowledge in addition to the physical and emotional challenges of childcare [14].

However, support, challenges and needs among caregivers of children with special needs likely vary in different geographical settings. Few such studies have been conducted in Eastern Europe. We hypothesized that the majority of parents and caregivers of CDD in Croatia, North Macedonia and Serbia will indicate that they are not satisfied with the level of support for a child, that they had difficulties with the availability of services for a child, and that their greatest challenge will be making sure their child receives adequate health care.

This study aimed to analyze the perceptions of support, challenges and needs among caregivers of children with developmental disabilities in Croatia, North Macedonia and Serbia.

## Methods

### Study design

This was a cross-sectional study. Data were collected via an online survey.

### Ethics

The Ethics Committee of the Catholic University of Croatia approved the study protocol (Classification number 641-03/23 − 03/045; Registration number: 498-15-06-23-002; issued on March 3, 2023). All methods were performed in accordance with the relevant guidelines and regulations. Prospective participants received detailed information about the study with the invitation to participate. Informed consent was obtained from the participants prior to the survey via the online survey interface.

### Reporting

The study was reported in line with the Checklist for Reporting Results of Internet E-Surveys (CHERRIES) [15].

### Participants

Inclusion criteria: The eligible participants were caregivers of CDD in Croatia, North Macedonia and Serbia. Caregivers were defined as any person with parenting or caring responsibility for CDD up to the age of 18, who could be in hospital or at home [16].

CDD were defined as children who have or are at increased risk of chronic physical, developmental, behavioral or emotional conditions and who require healthcare and related services of a type or amount beyond that required by children generally [1].

Exclusion criteria: We excluded individuals whose children suffered from conditions that are not defined as CDD.

Potential participants were contacted via associations for parents/caregivers of children with special needs. To get wider distribution, recipients of the invitation were encouraged to invite other eligible individuals to take part in the survey in a variation of snowball sampling.

The participants were invited via e-mail and social networks using the invitation provided in Supplementary file 1. The invitation contained a detailed information sheet about the study and a link to the online survey. Potential participants received two reminders spaced two weeks apart. Participants did not receive any financial or non-financial incentives. Participation in the study was voluntary and anonymous.

### Survey

For this study, we used a survey based on the Caregiver Needs Survey, which was developed by Amy Daniels and National Coordinators of The Southeast European Autism Network (SEAN), as a part of the Global Autism Public Health Initiative of the organization Autism Speaks (https://www.autismspeaks.org/*).* The Caregiver Needs Survey was previously used in Serbia [17]. The full text of the survey in English is available in Supplementary file 2.

Our survey analyzed the following: demographic characteristics; affected child characteristics, including whether a child suffers from chronic pain, child’s dietary habits and physical activity; service encounters and parent/caregiver perceptions. A detailed description of modifications we made to the Caregiver Needs Survey is provided in Supplementary file 3. Data on pain, nutrition and physical activity were not related to this study aim, so they were not presented in this manuscript.

Since the survey was targeting participants from three different countries, we created three separate surveys in SurveyMonkey for the Croatian, Macedonian and Serbian languages (surveys in all three languages are available on the Open Science Framework page for this project; link: https://osf.io/chxyr/).

The survey was shown to the participants on 26 pages in SurveyMonkey. We used skip logic in the survey to present different follow-up questions based on participants’ responses. Participants were able to review and change their responses by going back to the prior page of the survey. We did not use cookies to assign a unique user identifier to each device. The survey did not collect data about the participants’ names or e-mail addresses. Information about the IP address was not collected. We did not use any techniques to try to prevent duplicate entries. Participants were not asked for registration. The survey was not password-protected.

### Development and testing of the survey

The survey was developed in SurveyMonkey and pilot-tested on a sample of 5 individuals who fulfilled the inclusion criteria. Pilot testers assessed the usability and technical functionality of the electronic survey. Pilot testers were not included in the main study sample because we modified several questions and answers based on their feedback and suggestions for clarification.

### Data analysis

We analyzed all surveys, regardless of the incomplete answers and early terminations. We did not exclude any surveys based on the time spent in the online survey interface. We did not make any corrections or adjustments to the raw data.

The survey data were extracted from SurveyMonkey and analyzed using descriptive statistics in Microsoft Excel. Raw data were published on the Open Science Framework without indirect identifiers upon survey completion. We planned to censor any identifying data provided as part of free-text answers, but there were no such identifying details in the responses to open answers.

For continuous data, we used the Kolmogorov-Smirnov test to analyze the normality of distribution. None of the continuous data were normally distributed. We presented those data as the median and interquartile range (IQR). In the survey, we asked participants to report their ages as years and months. But in the analyses, we analysed their responses as years. Differences among countries in the frequency of categorical and discrete variables were analyzed using chi-squared test. Differences between countries in continuous variables were tested using the Kruskal-Wallis test.

The open-ended questions were analyzed using a qualitative description (QD) method [18]. QD involves making a descriptive summary of the data collected without an attempt to reinterpret the participants’ comments. This contrasts with other qualitative approaches that draw new insights and/or theories from the data, including grounded theory and phenomenology. We were able to explain and arrange our findings using qualitative description since our qualitative data consisted only of succinct, open-ended statements, allowing us to avoid trying to reinterpret the comments made by participants. To begin with, qualitative responses were reviewed and coded. Two authors were involved in the coding of the qualitative content. One author suggested the codes (AC), and another author verified it (LP). Coding definitions were applied to the data through an iterative process, until a consensus was achieved about the final coding categories. The process was repeated until all authors involved in this analyses agreed on how the codes were applied to the data.

### Data availability

The raw data generated in the study are available on Open Science Framework (link: https://osf.io/chxyr/) and made publicly accessible, except for the demographic information, i.e., indirect identifiers. The invited participants were informed about this in the information sheet that was sent with the invitation to participate in the study.

## Results

In total, 954 parents/caregivers filled out the survey. We excluded one survey because it reported that the child suffered from schizophrenia. We included 953 surveys in the analysis, 542 (57%) from Croatia, 205 (21%) from North Macedonia and 206 (22%) from Serbia.

### Participants’ characteristics

Most participants had a secondary school level of education (*N* = 536; 56.18%), and their partner/spouse also most often had a secondary school level of education (*N* = 572; 64.85%). The median age of the participants was 38 years; the median age of the spouse/partner was 40 years. The median number of children in the family was 2. The median number of children with developmental disabilities in the family was 1 (Table 1).

**Table 1:** Participants’ characteristics

Variable	Croatia	North Macedonia	Serbia	Total	*p*
Highest level of education, *N* (%)					
- Primary school - Secondary school - University degree - MSc/PhD	45 (8.29) 372 (68.69) 114 (20.99) 11 (2.03)	10 (4.88) 74 (36.10) 93 (45.37) 28 (13.66)	8 (3.88) 89 (43.20) 92 (44.66) 17 (8.25)	63 (6.60) 535 (56.18) 299 (31.34) 56 (5.88)	0.499 < 0.001 < 0.001 < 0.001
**Age, median (IQR)**	38 (10)	41 (9)	38 (8)	38 (9)	< 0.001*^1^
**Spouse’s/partner’s highest level of education, N (%)**					
- Primary school - Secondary school - University degree - MSc/PhD	45 (9.05) 376 (75.65) 62 (12.68) 13 (2.62)	10 (5.15) 99 (51.03) 72 (37.11) 13 (6.70)	9 (4.71) 97 (50.79) 71 (37.17) 14 (7.33)	64 (7.26) 572 (64.85) 205 (23.36) 40 (4.53)	0.78 < 0.001 < 0.001 < 0.001
**Age of spouse/partner, median (IQR)**	40 (9.5)	43 (10)	40 (10)	41 (10)	< 0.001*^2^
**Number of children, median (IQR)**	2 (1)	2 (0)	2 (1)	2 (1)	NS
**Number of children with developmental disabilities, median (IQR)**	1 (0)	1 (0)	1 (0)	1 (0)	NS

Acronyms: IQR = interquartile range, NS = not significant

*p-values for Kruskal-Wallis test; the remaining *p*-values refer to chi-square test

^1^the Post-Hoc Dunn’s test using a Bonferroni corrected alpha of 0.017 indicated that the mean ranks of the following pairs are significantly different: Croatia and North Macedonia, North Macedonia and Serbia

^2^the Post-Hoc Dunn’s test using a Bonferroni corrected alpha of 0.017 indicated that the mean ranks of the following pairs are significantly different: Croatia and North Macedonia, North Macedonia and Serbia

### Child characteristics

The most common diagnosis of CDD taken care of by the participants was autism spectrum disorder, observed in 26% of children (Supplementary Table 1 in Supplementary file 4). The majority of children were male (*N* = 508; 69.40%), and their current median age was 7 years.

Children had a median of 2 years when the participants had first concerns about the child’s development. The most common first concerns that the participants had were child’s problems with fine motor skills, such as using scissors or drawing with crayons (*N* = 310; 42.35%), followed with having difficulties in playing/interacting with others (*N* = 300; 40.99%) and not making eye contact when talking or playing with others (*N* = 276; 37.70%). However, we found some differences between the three countries in first concerns: most caregivers in Serbia firstly noticed not making eye contact, in Croatia problems with fine motor skills while in North Macedonia caregivers were mostly worried because children did not respond to sounds (Table 1). Among other concerns that the participants mentioned, the most common concerns were mental disorders and other behavioral disorders (*N* = 44; 4.61%) (Supplementary Table 2 in Supplementary file 4). The child most often received the first diagnosis at the median of 2 years and was most often diagnosed by a team of professionals (*N* = 217; 29.64%) (Table 2). There were some differences among countries. Namely, in Serbia most diagnoses were established by psychiatrists, in North Macedonia by neurologists, and in Croatia by the team of proffesionals and by pediatricians (Table 2). Among “other” individuals who diagnosed a child, the most common were speech therapists (*N* = 22; 2.30%) (Supplementary Table 3 in Supplementary file 4).

**Table 2:** Characteristics of participants’ children with developmental disabilities

Variable	*N* (%)				*p*
	Croatia	North Macedonia	Serbia	Total	
Child’s gender, *N* (%)					
Male Female	262 (67.26) 128 (32.74)	121 (70.76) 50 (29.24)	124 (72.94) 46 (27.06)	507 (69.40) 224 (30.60)	0.97 0.88
**Child’s current age, median (IQR)**	8 (7)	9 (8)	5 (4)	7 (8)	< 0.001*^1^
**How old was your child when you first had a concern about the child’s development, median (IQR)**	1 (2)	2 (2)	1.5 (1)	2 (2)	NS
**First concerns that the participants had about their children (multiple answer were allowed)**:					
Had medical problems such as seizures, lack of physical growth, or stomach problems Didn’t make eye contact when talking or playing with others Didn’t respond when called or didn’t respond to sounds Didn’t seem to understand nonverbal communication, such as understanding what you meant by the tone of voice you used or your facial expressions or other body language cues Had behavioral difficulties such as sleeping or eating problems, high activity level, wandering, tantrums, aggressive or destructive behavior Had problems with coordination or gross motor skills, such as walking Talked later than usual for most children Was not talking at all Did not talk as well as other children that were the same age Some speech skills that he/she had already developed were lost Didn’t seem to understand what you or other adults said to him/her Had problems with fine motor skills, such as using scissors or drawing with crayons Had difficulty playing or interacting with others Had difficulty learning new skills such as toilet training or getting dressed Had difficulty learning new things, such as the alphabet or numbers Other	78 (19.95) 130 (33.25) 106 (27.11) 104 (26.60) 132 (33.76) 127 (32.48) 127 (32.48) 105 (26.85) 99 (25.32) 84 (21.48) 118 (30.18) 176 (45.01) 161 (41.18) 123 (31.46) 102 (26.09) 81 (20.72)	29 (16.96) 56 (32.75) 75 (43.86) 60 (35.09) 51 (29.82) 59 (34.50) 34 (19.88) 71 (41.52) 46 (26.90) 45 (26.32) 56 (32.75) 73 (42.69) 70 (40.94) 53 (30.99) 41 (23.98) 42 (24.56)	22 (12.94) 90 (52.94) 79 (46.47) 62 (36.47) 45 (26.47) 37 (21.76) 36 (21.18) 60 (35.29) 54 (31.76) 48 (28.24) 82 (48.24) 61 (35.88) 69 (40.59) 57 (33.53) 23 (13.53) 37 (21.76)	129 (17.62) 276 (37.70) 260 (35.52) 226 (30.47) 228 (31.14) 223 (30.47) 197 (26.91) 236 (32.24) 199 (27.19) 177 (24.18) 256 (34.97) 310 (42.35) 300 (40.99) 233 (31.83) 166 (22.68) 160 (21.86)	< 0.001 < 0.001 < 0.001 < 0.001 0.723 < 0.001 < 0.001 < 0.001 0.475 0.018 < 0.001 < 0.001 0.701 0.319 0.026 0.171
**The age of the child when they were first diagnosed with a developmental disability, median (IQR)**	3 (3)	3 (2)	2 (2)	2 (3)	NS
**Who determined the child’s initial diagnosis**					
- Primary care physician/Family physician - Pediatrician - Pediatric specialist (i.e. developmental pediatrician) - Psychologist - Psychiatrist - Neurologist - Nurse - Team of professionals - Other	2 (0.51) 29 (7.42) 115 (29.41) 28 (7.16) 20 (5.12) 19 (4.86) 0 (0.00) 124 (31.71) 54 (13.81)	6 (3.51) 7 (4.09) 17 (9.94) 3 (1.75) 10 (5.85) 56 (32.75) 0 (0.00) 54 (31.58) 18 (10.53)	2 (1.18) 3 (1.76) 9 (5.29) 17 (10.00) 62 (36.47) 19 (11.18) 0 (0.00) 39 (22.94) 19 (11.18)	10 (1.37) 39 (3.98) 141 (19.27) 48 (6.56) 92 (12.57) 94 (12.90) 0 (0.00) 217 (29.64) 91 (12.43)	0.009 0.048 < 0.001 0.006 < 0.001 < 0.001 - < 0.001 0.872

Acronym: IQR = interquartile range, NS = not sigificant

*p-values for Kruskal-Wallis test; the remaining *p*-values refer to chi-square test

^1^the Post-Hoc Dunn’s test using a Bonferroni corrected alpha of 0.017 indicated that the mean ranks of the following pairs are significantly different: Croatia and Serbia, North Macedonia and Serbia

### Service encounters

Most of the children in our study attended preschool (29%) and public school (29%), or 58% in total. Only 22% of the children were not attending any form of schooling. There were 391 (57.25%) children who received additional academic support due to their developmental disabilities. There were 449 (65.74%) children that received assistance from the state/city/county. Most commonly, they received help from the state (*N* = 355; 79.78%) (Table 3), especially in North Macedonia (88.79%). Other mentioned sources of assistance were various associations and donations (Supplementary Table 4 in Supplementary file 4). Most participants declared they do not participate in associations or organizations for family support (*N* = 492; 72.78%). Participants used multiple sources of information about their child’s condition, mostly experts (i.e., health specialists, therapists) who work with a child (*N* = 473; 67.97%), followed by the Internet (*N* = 357; 52.81%) (Table 3). The answers were similar in all countries; the differences were found in Croatia that caregivers more used teachers as a source of information (34.87% vs. 22% in Serbia and North Macedonia) and in using experts in North Macedonia (only 58.28%).

**Table 3:** Education or other services or treatments that child may have received in the past or is currently receiving to meet their needs

Questions	*N* (%)				p*
	Croatia	North Macedonia	Serbia	Total	
What kind of school is your child currently enrolled in?					
- Preschool - Public school - Private school - Special school for children with disabilities - Not enrolled in preschool/school	97 (27.64) 120 (34.19) 1 (0.28) 63 (17.95) 70 (19.94)	34 (20.48) 62 (37.35) 3 (1.81) 33 (19.88) 34 (20.48)	70 (42.17) 16 (9.64) 1 (0.60) 34 (20.48) 45 (27.11)	201 (29.43) 198 (28.99) 5 (0.73) 130 (19.03) 149 (21.82)	< 0.001 < 0.001 < 0.001 < 0.001 0.010
**Does your child receive any additional academic support because of his/her developmental needs?**					
- Yes - No	205 (58.40) 146 (41.60)	96 (57.83) 70 (42.17)	90 (54.22) 76 (45.78)	391 (57.25) 292 (42.75)	0.056 0.060
**Does your child currently receive any special assistance from the government/city/municipality etc., because of his/her developmental disability?**					
- Yes - No	225 (64.10) 126 (35.90)	109 (65.66) 57 (34.34)	115 (69.28) 51 (30.72)	449 (65.74) 234 (34.26)	0.061 0.055
**Please indicate what special assistance you receive (multiple answers were allowed)**:					
- Help from the state - Help from the city - Help from the county - Help from a religious organization - Some other help	170 (76.23) 57 (25.56) 12 (5.38) 0 (0.00) 29 (13.00)	95 (88.79) 3 (2.80) 9 (8.41) 1 (0.93) 16 (14.95)	90 (78.26) 8 (6.96) 8 (6.96) 0 (0.00) 23 (20.00)	355 (79.78) 68 (15.28) 146 (32.80) 1 (0.22) 68 (15.28)	< 0.001 < 0.001 0.068 **-** 0.020
**Do you or any family member currently participate in any family support, advocacy group or organization because of the child’s developmental disability?**					
- Yes - No	109 (31.41) 238 (68.59)	30 (18.40) 133 (81.60)	45 (27.11) 121 (72.89)	184 (27.22) 492 (72.78)	0.138 < 0.001
**To what source(s) do you typically turn to get information about your child’s condition (multiple answer were allowed)?**					
- The Internet - My child’s primary care physician/pediatrician - My child’s teacher - Other parents of children with developmental disabilities - Experts (i.e. health specialists, therapists) who work with my child) - Other	190 (54.76) 169 (48.70) 121 (34.87) 178 (51.30) 245 (70.61) 9 (2.59)	87 (53.37) 78 (47.85) 36 (22.09) 78 (47.85) 95 (58.28) 13 (7.98)	80 (48.19) 72 (43.37) 38 (22.89) 77 (46.39) 133 (80.12) 14 (8.43)	357 (52.81) 319 (47.19) 195 (28.85) 333 (49.26) 473 (67.97) 36 (5.32)	0.562 0.181 < 0.001 0.205 < 0.001 < 0.001

*p-values for chi-square test

### Parent/caregiver perceptions

In the last section related to access and unmet needs, most participants stated that they did not experience difficulties or delays in getting services for their child in the last 12 months (*N* = 378; 57.10%) (Table 4). Some parents indicated that the provision of services was inadequate or discriminatory (*N* = 36; 3.77%) (Supplementary Table 5 in Supplementary file 4). Most participants indicated that in the last 12 months, they had difficulties with the availability of services because services were unavailable in the area or due to waiting lists (*N* = 397; 59.97%). In the last 12 months, most participants did not have difficulty paying for services (*N* = 411; 62.08%). Most of them did not have problems getting the necessary information (*N* = 355; 53.63%) (Table 4).

**Table 4:** Types of services children may need or use

Questions	*N* (%)				p*
	Croatia	North Macedonia	Serbia	Total	
**During the past 12 months, did you have any difficulties or delays in getting services for your child because the child was not eligible for the services?**					
- Yes - No	148 (44.05) 188 (55.95)	75 (46.58) 86 (53.42)	61 (36.97) 104 (63.03)	284 (42.90) 378 (57.10)	0.467 0.562
**During the past 12 months, did you have any difficulties or delays because services the child needed were not available in your area?**					
- Yes - No	167 (49.70) 169 (50.30)	80 (49.69) 81 (50.31)	95 (57.58) 70 (42.42)	342 (51.66) 320 (48.34)	0.187 0.462
**During the past 12 months, did you have any difficulties or delays because there were waiting lists, backlogs, or other problems getting appointments?**					
- Yes - No	209 (62.20) 127 (37.80)	93 (57.76) 68 (42.24)	95 (57.58) 70 (42.42)	397 (59.97) 265 (40.03)	0.677 0.267
**During the past 12 months, did you have any difficulties or delays because you couldn’t pay for it?**					
- Yes - No	126 (37.50) 210 (62.50)	56 (34.78) 105 (65.22)	69 (41.82) 96 (58.18)	251 (37.92) 411 (62.08)	0.827 0.004
**During the past 12 months, did you have any difficulties or delays because you had trouble getting the information you needed?**					
- Yes - No	152 (45.24) 184 (54.76)	75 (46.58) 86 (53.42)	80 (48.48) 85 (51.52)	307 (46.37) 355 (53.63)	0.029 0.117
**During the past 12 months, did you have any difficulties or delays for any other reason?**					
- Yes - No	78 (23.21) 258 (76.79)	54 (33.54) 107 (66.46)	57 (34.55) 108 (65.45)	189 (28.55) 473 (71.45)	< 0.001 < 0.001
**Please, could you describe those other reasons?**					
**During the past 12 months, how often have you been frustrated in your efforts to get services for your child?**					
- Never - Sometimes - Usually - Always	39 (12.00) 164 (50.46) 58 (17.85) 64 (19.69)	78 (49.06) 46 (28.93) 0 (0) 35 (22.01)	24 (15.00) 70 (43.75) 33 (20.63) 33 (20.63)	141 (21.89) 280(43.48) 91 (14.13) 132 (20.50)	< 0.001 < 0.001 0.105
**Has your child’s condition caused financial problems for your family?**					
- Yes - No	126 (50.81) 122 (49.19)	95 (76.00) 30 (24.00)	91 (66.42) 46 (35.58)	312 (61.18) 198 (38.82)	< 0.001 < 0.001
**Have you or other family members stopped working because of your child’s condition?**					
- Yes - No	148 (59.58) 100 (40.32)	58 (46.40) 67 (53.60)	76 (55.47) 61 (44.53)	282 (55.30) 228 (44.70)	< 0.001 < 0.001
**Have you or other family members cut down on the hours your work because of your child’s condition?**					
- Yes - No	117 (47.18) 131 (52.82)	68 (54.40) 57 (45.60)	69 (50.36) 68 (49.64)	254 (49.80) 256 (50.20)	< 0.001 < 0.001
**What you consider to be the greatest challenges in caring for a child with developmental disability (three answer were allowed)**:					
- Challenging behaviors (i.e. self-injury, aggression, tantrums) - Daily living skills (i.e. toileting, self-feeding, self-care) - Health problems (i.e. co-occurring physical and/or mental health conditions) - Sleep problems (i.e. trouble falling asleep, trouble staying asleep) - Diet/eating/feeding difficulties - Social interaction difficulties - Repetitive behaviors/restrictive interests - Communication difficulties - Safety concerns (i.e. wandering, climbing) - Sensory issues (sensitivity on sounds, food textures, touch etc.) - Other	69 (27.82) 117 (47.18) 90 (36.29) 41 (16.53) 74 (29.84) 135 (54.44) 66 (26.61) 143 (57.66) 49 (19.76) 80 (32.26) 16 (6.45)	40 (32.00) 79 (63.20) 49 (39.20) 23 (18.40) 26 (20.80) 74 (59.20) 29 (23.20) 77 (61.60) 21 (16.80) 30 (24.00) 21 (16.80)	41 (29.93) 68 (49.64) 44 (32.12) 31 (22.63) 31 (22.63) 76 (55.47) 22 (16.06) 88 (64.23) 18 (13.14) 27 (19.71) 26 (18.98)	150 (29.41) 264 (51.76) 183 (35.88) 95 (18.62) 131 (25.68) 285 (55.88) 117 (22.94) 308 (60.39) 88 (17.25) 137 (26.86) 63 (12.35)	0.013 < 0.001 0.525 < 0.001 0.781 0.681 0.560 0.577 0.671 < 0.001 < 0.001
**The greatest challenges you face in getting support for your child (three answer were allowed)?**					
- Making sure my child receives adequate health care - Making sure my child receives adequate education - Making sure my child receives adequate welfare / social supports - Making sure my child’s basic rights are protected - Making sure my family and I receive adequate respite - Other	173 (69.76) 165 (66.53) 152 (61.29) 198 (79.84) 104 (41.94) 11 (4.44)	98 (78.40) 89 (71.20) 83 (66.40) 89 (71.20) 40 (32.00) 20 (16.00)	92 (67.15) 92 (67.15) 90 (65.69) 91 (66.42) 67 (48.91) 14 (10.22)	363 (71.18) 346 (67.84) 325 (63.72) 378 (74.11) 211 (41.37 45 (8.82)	< 0.001 < 0.001 < 0.001 < 0.001 < 0.001 < 0.001
**What you consider to be the greatest priorities for affected families in your country (three answer were allowed)?**					
- Improved health care services - Improved education services - Improved welfare / social services - Greater rights for individuals with developmental disabilities - Greater protection of existing rights for individuals with developmental disabilities - More information about people with developmental disabilities - Greater in-home support - Greater community awareness - Greater opportunities for parent interactions / networking - Providing assistance to healthy children in the family to better cope with problems related to the children with developmental disabilities - Provision of psychological help for parents - A greater number of Institutions/Centers for working with children with developmental disabilities - More professional staff in existing institutions in the place where the child lives - Availability of associations for parents of children with developmental disabilities in the place where the child lives - Better education of existing experts to notice symptoms of developmental delays - Better education for parents of newly diagnosed children with developmental disabilities in order to teach them how to work with their children - Other	142 (57.26) 114 (45.97) 101 (40.73) 180 (72.58) 95 (38.31) 78 (31.45) 47 (18.95) 98 (39.52) 40 (16.13) 66 (26.61) 76 (30.65) 100 (40.32) 92 (37.10) 48 (19.35) 78 (31.45) 84 (33.87) 8 (3.23)	70 (56.00) 59 (47.20) 54 (43.20) 63 (50.40) 43 (34.40) 37 (29.60) 28 (22.40) 53 (42.40) 17 (13.60) 23 (18.40) 37 (29.60) 71 (56.80) 57 (45.60) 31 (24.80) 39 (31.20) 43 (34.40) 13 (10.40)	68 (49.64) 49 (35.77) 46 (33.58) 62 (45.26) 33 (24.09) 38 (27.74) 31 (22.63) 51 (37.23) 17 (12.41) 25 (18.25) 45 (32.85) 71 (51.82) 63 (45.99) 19 (13.87) 38 (27.74) 38 (27.74) 15 (10.95)	280 (54.90) 222 (43.53) 201 (39.41) 305 (59.80) 171 (33.53) 153 (30.00) 106 (20.78) 202 (39.60) 74 (14.50) 114 (22.35) 158 (30.98) 242 (47.45) 212 (41.57) 98 (19.215) 120 (23.52) 165 (32.35) 36 (7.06)	0.451 0.571 0.011 0.650 0.363 0.568 0.671 0.560 0.456 0.933 0.456 < 0.001 < 0.001 < 0.001 0.657 0.195 < 0.001

*p-values for chi-square test

During the past 12 months, most participants sometimes had frustrations about trying to get child services (*N* = 280; 42.48%), although almost half of the Macedonian caregivers reported that they never had these frustrations. Most participants indicated that the child’s condition cused financial problems (*N* = 312; 61.18%), especially in North Macedonia (76.00%). Most participants or other family members stopped working because of their child’s condition (*N* = 282; 55.30%), (59.58% in Croatia), while half of them reduced their working hours (*N* = 254; 49.80%). In all countries participants stated communication difficulties as the main problem in care (*N* = 308; 60.39%), followed by social interacions The biggest problem in getting support for the child was ensuring the child’s basic rights were protected (*N* = 378; 74.11%), although in Serbia and North Macedonia, caregivers were mostly worried about children’s healthcare and education. Participants stated that ensuring greater rights for individuals with developmental disabilities (*N* = 305; 59.80%) was the greatest need for families of children with developmental disabilities (72.58% answers in Croatia). In addition, caregivers in Serbia and North Macedonia pointed out the need for improved health care services (49.64% and 56.00% respectively) and the need for a greater number of Institutions/Centers for working with children with developmental disabilities (51.82% and 56.80% respectively, Table 4).

When asked if they feel helpless because they have a CDD, most gave a neutral response (*N* = 167; 32.75%). When asked if they worry about whether other people will know that they have a child with developmental disabilities, most participants disagreed (*N* = 157; 30.78%). When asked if other people would discriminate against them because they have a child with developmental disabilities, most participants disagreed (*N* = 39; 28.47%). Most strongly disagreed or disagreed (*N* = 310; 60.77%) with the statement that having a child with a developmental disability impacts them negatively (Table 5).

**Table 5:** Parents’ level of agreement with statements about feeling helpless and worries about having a child with developmental disabilities

Statement	*N* (%)				
	Strongly disagree	Disagree	Neither agree nor disagree	Agree	Strongly agree
I feel helpless for having a child with a developmental disability					
**Croatia** **North Macedonia** **Serbia** **Total**	44 (17.74) 20 (16.00) 21 (15.33) 85 (16.66)	49 (19.76) 19 (15.20) 34 (24.82) 102 (20.00)	77 (31.05) 42 (33.60) 48 (35.04) 167 (32.75)	58 (23.39) 40 (32.00) 25 (18.25) 123 (24.11)	20 (8.06) 4 (3.20) 9 (6.57) 33 (6.47)
I worry if other people would know I have a child with a developmental disability					
**Croatia** **North Macedonia** **Serbia** **Total**	115 (46.37) 55 (44.00) 64 (46.72) 234 (45.88)	86 (34.68) 27 (21.60) 44 (32.12) 157 (30.78)	24 (9.68) 19 (15.20) 19 (13.87) 62 (12.15)	19 (7.66) 11 (8.80) 9 (6.57) 39 (7.64)	4 (1.61) 13 (10.40) 1 (0.73) 18 (3.52)
Other people would discriminate against me because I have a child with a developmental disability					
**Croatia** **North Macedonia** **Serbia** **Total**	74 (29.84) 35 (28.00) 36 (26.28) 146 (28.62)	69 (27.82) 20 (16.00) 39 (28.47) 128 (25.10)	68 (27.42) 35 (28.00) 45(32.85) 148 (29.01)	32 (12.90) 29 (23.20) 16 (11.68) 77 (15.10)	5 (2.02) 6 (4.80) 1 (0.73) 12 (2.35)
Having a child with a developmental disability imposes a negative impact on me					
**Croatia** **North Macedonia** **Serbia** **Total**	84 (33.87) 24 (19.20) 36 (26.28) 144 (28.23)	92 (37.10) 35 (28.00) 39 (28.47) 166 (32.54)	49 (19.76) 33 (26.40) 34 (24.82) 116 (22.74)	21 (8.47) 27 (21.60) 23 (16.79) 71 (13.92)	2 (0.81) 6 (4.80) 5 (3.65) 13 (2.54)

Most participants were satisfied with the support of educators, teachers and professional services. Also, most participants were satisfied with the support that a child has in making progress at home. Most participants were neutral regarding the support the child has for making friends. Most participants were satisfied with the providers who work with the child. Most participants were satisfied with the support of family and friends (Table 6).

**Table 6:** Participants’ satisfaction with the level of support for a child with developmental disabilities

Statements	*N* (%)				
	Very dissatisfied	Dissatisfied	Neither dissatisfied nor satisfied	Satisfied	Very satisfied
Support for my child by the teachers and school team of experts other than teachers (i.e. psychologist, occupational therapist, speech therapist to make progress in kindergarten/school					
**Croatia** **North Macedonia** **Serbia** **Total**	26 (10.48) 6 (4.80) 9 (6.57) 41 (8.03)	30 (12.10) 25 (20.00) 12 (8.76) 67 (13.13)	56 (22.58) 46 (36.80) 38 (27.74) 140 (27.45)	89 (35.89) 29 (23.20) 52 (37.96) 170 (33.33)	47 (18.95) 19 (15.20) 26 (18.98) 92 (18.03)
Support for my child to make progress at home					
**Croatia** **North Macedonia** **Serbia** **Total**	15 (6.05) 5 (4.00) 11 (8.03) 31 (6.08)	25 (10.08) 25 (20.00) 17 (12.41) 67 (13.13)	69 (27.82) 33 (26.40) 34 (24.82) 136 (26.66)	98 (39.52) 41 (32.80) 50 (36.50) 189 (37.06)	41 (16.53) 21 (16.80) 25 (18.25) 87 (17.06)
Support for my child to make friends					
**Croatia** **North Macedonia** **Serbia** **Total**	31 (12.50) 17 (13.60) 7 (5.11) 55 (10.78)	39 (15.73) 36 (28.80) 28 (20.44) 103 (20.20)	78 (31.45) 37 (29.60) 59 (43.07) 174 (34.11)	86 (34.68) 29 (23.20) 35 (25.55) 150 (29.41)	14 (5.65) 6 (4.80) 8 (5.84) 28 (5.50)
Relationship with the service providers (speech therapist, psychologists, special rehabilitator and educator) who work with my child					
**Croatia** **North Macedonia** **Serbia** **Total**	10 (4.03) 3 (2.40) 3 (4.38) 16 (3.13)	20 (8.06) 9 (7.20) 7 (5.11) 36 (7.06)	45 (18.15) 29 (23.20) 28 (20.44) 102 (20.00)	117 (47.18) 57 (45.60) 61 (44.53) 235 (46.08)	56 (22.58) 27 (21.60) 38 (27.74) 121 (23.72)
Support from your friend					
**Croatia** **North Macedonia** **Serbia** **Total**	12 (4.84) 4 (3.20) 6 (4.38) 22 (4.31)	28 (11.29) 16 (12.80) 17 (12.41) 61 (11.96)	62 (25.00) 42 (33.60) 34 (24.82) 138 (27.06)	100 (40.32) 47 (37.60) 60 (43.80) 207 (40.59)	46 (18.55) 16 (12.80) 20 (14.60) 82 (16.08)
Support from your family					
**Croatia** **North Macedonia** **Serbia** **Total**	12 (4.84) 4 (3.20) 5 (3.65) 21 (4.12)	25 (10.08) 8 (6.40) 10 (7.30) 43 (8.43)	59 (23.79) 25 (20.00) 23 (16.79) 107 (20.98)	96 (38.71) 53 (42.40) 52 (37.96) 201 (39.41)	56 (22.58) 35 (28.00) 47 (34.31) 138 (27.06)

For some open-ended questions in our survey, we did not get any answers that we could categorize. Those questions are listed in Supplementary Table 6 in Supplementary file 4.

## Discussion

Our study provided results about the current support, challenges and needs of parents and caregivers of CDD in Croatia, North Macedonia and Serbia. The results were comparable between the three countries, indicating that the parents and children in the region share the same challenges and needs, and require the same support.

Pejović-Milovančević et al. conducted a study on children affected with autism in Serbia in 2016 and 2017 on a sample of 231 parents. They found that almost 40% of children were enrolled in regular preschool or school, which attests to the effort to include those children in the regular school system [17]. In our study, 58% of children attended preschool or public school. The different sample may explain the differences, as our study did not include only children with autism.

The 2015 Education For All (EFA) Global Monitoring analysis, conducted across 30 countries hosting Plan International sponsorship programs, found that children with disabilities were far less likely to attend school, had less accumulated schooling and were more likely to report a serious illness in the last year. Furthermore, children with hearing or visual impairments had better schooling outcomes compared with children with learning or communication impairments [19].

In Europe, all countries have inclusive education as a policy vision, but the countries implement this vision in different ways. For example, Member States of the European Union (EU) have different laws, policies and systems, particularly for education. A report prepared by the European Agency for Special Needs and Inclusive Education, and published in 2017, showed that in 2012/2013 across the 28 EU countries, the enrolment rate in mainstream education ranged from 93.44 to 99.88%. All those countries have legislation requiring all children, including CDD, to attend some form of schooling. However, despite such legislative requirements, there are still CDD that are not enrolled in any form of schooling, or are not attending schooling regularly [20]. Data for Croatia were available in that report. North Macedonia and Serbia are not EU member states. Data from our study cannot be directly compared with the data published by the European Agency for Special Needs and Inclusive Education because we did not limit our inclusion criteria to children that were of school age.

In our study, most of the caregivers had the first concerns about the child’s development at the median age of 2, as problems with fine motor skills (such as using scissors or drawing with crayons), followed by difficulty in playing or interacting with other, not making eye contact when talking or playing with others, and not responding when called or to sounds, although some differences in percentages of the caregivers’ first concerns were found among the countries. Similarly, the majority of the parents who have children with fragile X syndrome (FXS) phenotype reported initial concerns prior to the child’s first birthday and in most cases, it was deviant motor behaviors [21]. In children with autism, the first concern included problems of interacting with others or playing alone, unusual gestures or movements, and the child not understanding what parents or other adults said to him/her [17].

It is worth emphasizing that the duration between the first concerns of the parents and families and receiving a medical diagnosis in this study was very commendable, and not even seen in many developed countries [22]. Good practices in Croatia, North Macedonia and Serbia that aided the duration between the first concerns of the parents and families and receiving a medical diagnosis could be due to the well-developed public health system. All children are assigned to a primary pediatrician, and they are expected to undergo regular preventive visits. This can aid in the timely diagnosis of developmental disorders.

In our study, the participants reported that the child’s initial diagnosis was determined by a team of professionals, followed by a pediatric specialist. Also, In North Macedonia and Serbia the diagnosis is often established by a neurologist or a psychiatrist, respectively. Early diagnosis is very important for starting early interventions. In 2023, Boulton et al. reported that in Sydney, Australia, the average age that caregivers identified developmental concerns was 3 years of age, but the average age of receiving a developmental assessment was 6.6 years. In that study, only 46% of children received a diagnostic assessment by 5 years of age, even though 88% of caregivers were concerned about their child’s development by that age [23]. Thus, we can conclude that the parents/caregivers’ concerns and diagnosis were timely in our sample.

The main sources of information about child’s condition for our participants were experts (i.e., health specialists, therapists) who worked with the child, followed by the Internet. Similarly, in the study by Stankovic et al., published in 2020, 20% of parents and caregivers of children with ADD sought information on how to treat a child during the COVID-19 pandemic from health care providers, while 13% sought help online [24].

Baumann et al. have reported that health information seeking does not differ significantly between parents with and without a disabled child. Their study, conducted in 2018, showed that despite the availability of digital media, personal contacts are still the most frequent health information resource for parents of young children, regardless of the child’s health [25].

In our study, most participants indicated that they were satisfied or very satisfied with the level of support for a child with developmental disabilities, and fewer than 25% of participants expressed dissatisfaction in this respect. In 2023, Lockwood Estrin et al. reported that in India, mothers of children with autism reported a perceived lack of family support, including from partners, and feeling unsupported, comparatively to the mothers of children with intellectual impairment, who described greater levels of perceived acceptance of developmental disabilities by family members, and also minimal impact on relationships between family members [26].

In 2017, Huus et al. reported that mothers of children with mild intellectual impairment in Sweden, with paid employment, were found to have a reduced need for support and that mothers with a higher education expressed fewer needs too. Also, they reported that there is a difference between the group with a child diagnosed only with mild intellectual impairment and the group with one or more additional diagnoses such as epilepsy, autism, Asperger’s syndrome, cerebral palsy, attention deficit hyperactivity disorder (ADHD) and developmental coordination disorder (DCD), and a speech or language disorder. Families of children with several diagnoses rated more needs for support and for community services [27].

Furthermore, in our study, most participants disagreed about feeling worried about whether other people will know that they have a child with developmental disabilities and feel discriminated because of it. Also, most disagreed with the statement that having a child with a developmental disability impacts them negatively.

In 2022, Niedbalski published the results of a qualitative study with parents of people with intellectual disabilities, focused on the context of disability stigma and pride. Parents talked about their children as a source of pride, describing positive social experiences, social relationships and interactions in the public sphere. However, parents also highlighted the painful and exhausting experience of dealing with various types of institutions, and thus, the negative role of stigma. Parents resisted the idea that their own lives should be framed simply in terms of tragedy, misfortune, or their child’s “deficits” [28].

In our study, most participants were satisfied with the support of educators, teachers and professional services. Also, most participants were satisfied with the support that the child has in making progress at home. Most participants were neutral regarding the support the child has for making friends. Most participants were satisfied with the providers who work with the child, as well as with the support of family and friends. A study of Lucic, conducted in Croatia in 4 waves from 2016 to 2019, found that parents of children with developmental difficulties, were less satisfied with health, relationships with family and friends, safety, and future security, compared to parents of typically developing children [29].

Multiple studies so far have pointed out the relevance of support from family and society, e.g., indicating that good relationships serve as a stress protector. The family’s support network of relatives, friends, and professional services may influence how the family copes with having a child with a disability. It has been reported that families with a strong support network have fewer needs [30, 31] and that improved social support might improve parenting efficacy [32].

The majority of participants indicated that in the last 12 months, they had difficulties with the availability of services in their area or problems getting appointments. This result is in accordance with the previous study conducted in Serbia during the COVID-19 pandemic, where 84.4% of children have not received any assistance or additional education relevant to their child’s needs while being at home [24]. Similarly, parents of children with autism often reported issues with service availability, long waiting lists for treatments, and lack of educational support, according to the lived experiences of parents of children with autism in Bosnia and Herzegovina [33].

Other reports from low- and middle-income countries indicate the need for enhanced services for CDD. For example, a study from Bulgaria, published in 2022, reported findings of a family needs assessment survey. They showed that children with ASD and those with other neurodevelopmental disabilities in Bulgaria had different needs, but that both experience similar problems in accessing medical, counseling, and educational services, regardless of their demographic characteristics. Parents indicated that their priorities were education, counseling, and medical support, protecting children’s basic rights, and raising awareness about the children’s needs [34].

A study from Indonesia indicated the need for government-run disability rehabilitation centers, the provision of fully subsidised health insurance, and the provision of qualified therapists and healthcare professionals [35].

In our study, reported difficulties with the availability of services and getting appointments could be perceived in contrast with the result that most of our participants indicated that they did not have difficulty paying for services. One potential explanation is that parents were unable to access public healthcare services, so they had to pay for private services.

Another contributing factor for limited access to services could possibly be due to a lack of specialists. It has already been described that there is a severe shortage of behavioral specialists who can assist children and families who are dealing with neurodevelopmental problems. Due to the lack of availability, services that can be delivered remotely have emerged. Telehealth is a method that can improve service accessibility, relieve budgetary burdens, and promote generalization assessments. It can promote evaluation and coaching using either synchronous or asynchronous components [36].

Kingsdorf and Pancocha have conducted a scoping review of recent behavioral telehealth practices for children and families impacted by neurodevelopmental disabilities in Europe [36]. Only six relevant publications were found to be included in the scoping review. The authors concluded that the need, empirical validation, and groundwork for the sustainability of behavioral telehealth practices already seem to exist, and that future work should focus on policies, procedures, and further research in this area [36].

The use of telehealth for CDD has increased since the start of the COVID-19 pandemic. However, different people will have different experiences, and limitations of the telehealth approach in children with CDD also need to be considered [37].

Of note, although the data came from three different countries, the findings were almost identical regarding the current diagnosis, issues, etc. This can be explained by the fact that these are neighbouring countries, which used to be part of the same country (former Yugoslavia), and they have similar health systems.

Limitations of the study could include potential misunderstandings of the survey questions. To ensure that the targeted participants will understand the questions, we conducted pilot testing before the beginning of the study, and we revised multiple questions based on the feedback of pilot-testers. However, it is possible that still some participants did not understand our questions the way they were intended. For example, one question asked, “*What do you consider to be the greatest priorities for affected families in your country?*”, and it is possible that the participants are not aware of challenges that could be applicable to the entire country. When asked about the child’s diagnosis, in addition to the name of the disability, some participants provided also information about comorbid conditions other than developmental disabilities (such as schizophrenia or anxiety).

Furthermore, we did not measure psychometric findings of the global developmental status or intellectual functioning of the participants. Thus, we do not have such data, which would be useful. Collecting such data could be considered in future studies on the topic.

Of note, our study was descriptive in nature. We did not hypothesize that we would expect differences between the subgroups per country or any other characteristics such as type of disability, and our study was not powered for such statistical analyses. Our study can be used to conduct such studies in future that will be fully powered for the hypothesized differences between the disability groups and that will explore analyses of the various associations of our findings with different variables.

## Conclusion

In conclusion, our study has identified unmet needs among parents and caregivers of CDD in Croatia, North Macedonia and Serbia. Parents/caregivers of CDD in Croatia, North Macedonia and Serbia faced multiple challenges, but most of them were satisfied with the services provided to their children. Future efforts to develop policies and services related to children with developmental disabilities should consider the opinions of their parents/caregivers and disparities in access to services.

### Electronic supplementary material

Below is the link to the electronic supplementary material.

Supplementary Material 1

Supplementary Material 2

Supplementary Material 3

Supplementary Material 4

## Data Availability

Raw data collected within the study (but without indirect identifiers of the participants) are available on Open Science Framework (link: https://osf.io/chxyr/) and made publicly accessible.
