# Supplementary material for: Perceptions on support, challenges and needs among parents and caregivers of children with developmental disabilities in Croatia, North Macedonia and Serbia: a cross-sectional study
Source: BMC Pediatr. 2024 May 3;24:297. doi: 10.1186/s12887-024-04770-7 (PMC11067112; doi:10.1186/s12887-024-04770-7)
Supplement: Supplementary file 1 — Supplementary Material 1 [file 12887_2024_4770_MOESM1_ESM.docx]

# **Supplementary file 1. E-mail invitation and information sheet for the prospective participants**

## ***E-mail invitation:***

**Title**: Invitation to take part in a study about support, challenges and needs among parents and caregivers of children with special needs

**Text of the e-mail:**

Dear colleague,

We invite you to participate in a study about support, challenges and needs among parents and caregivers of children with special needs.

The study is conducted via a brief online survey, which will take approximately 15 minutes to complete. The survey can be filled out on your mobile device or a desktop computer.

In the online survey, we will not collect your name or your email address. The results of this study will be used for research purposes only.

This survey was approved by the Ethics Committee of the Catholic University of Croatia. Please do not hesitate to contact the principal investigator, Prof. Livia Puljak (livia.puljak@unicath.hr), if you have any questions.

More detailed information about the study can be found in the information sheet at the bottom of this message.

To enter the survey, please use the following link: <https://www.surveymonkey.com/r/Q7R7PTX>

We would also appreciate it if you could forward this survey invitation to other parents and caregivers of children with special needs.

We are looking forward to your responses, and thank you in advance for your contribution.

Yours sincerely,

Prof. Livia Puljak, MD, PhD

Principal investigator

Head, Center for Evidence-Based Medicine

Catholic University of Croatia

e-mail: livia.puljak@unicath.hr

phone: + 385 (0) 1 370 66 33

## ***Information sheet (the attachment to the e-mail invitation)***

Study title: Support, challenges and needs among parents and caregivers of children with special needs

Dear Sir/Madam,

You are kindly invited to take part in a study about support, challenges and needs among parents and caregivers of children with special needs.

Before you decide about your participation, we would like to explain why this study is conducted and what it involves.

The aim of the study is to collect more information about the needs of parents/caregivers of children with special needs to better assist them.

The study is conducted via an online survey lasting no more than 15 minutes. The survey can be filled out on your mobile device or a desktop computer. If you decide to participate, on the first page of the survey, you will have the opportunity to express your consent to participate.

Participation in the study is voluntary. Nowhere in the survey will you be asked to provide your first and last name or your e-mail address. You can freely, without any consequences, withdraw from the survey at any time, without providing a reason. However, since we will not collect your name, email address or your IP address, we will not be able to remove any of your responses from our database.

The collected data will be used only for research and education purposes. We intend to publish a raw data set together with our data analysis, but without raw data about the participant demographics, to prevent their potential use as indirect identifiers.

The data will be stored in digital form on the computer of the principal investigator (Prof. Livia Puljak), protected by a password, for at least five years after the end of data collection. The researchers participating in the study will have access to the data during the research.

There are no risks associated with participation in this study; that is, the level of discomfort in this study is no greater than what you experience in everyday life situations. A potential benefit of participating in this study is that, based on the results, we could better help parents/caregivers of children with special needs in the future.

The study protocol was approved by the Ethics Committee of the Croatian Catholic University. The study is conducted in accordance with the guidelines for the safety of persons participating in this type of scientific research, including the Declaration of Helsinki.

If you would like to be informed about the results after the study has been completed, or if you have any questions regarding this study, please contact the principal investigator:

Prof. Livia Puljak, MD, PhD; e-mail: livia.puljak@unicath.hr; phone: + 385 (0) 1 370 66 33.

If you have any complaints regarding this study or how the data was collected, please contact the principal investigator:

Prof. Livia Puljak, MD, PhD; e-mail: livia.puljak@unicath.hr; phone: + 385 (0) 1 370 66 33.

Thank you for reading this document and considering participating in this study.

Prof. Livia Puljak, MD, PhD

Principal investigator

Head, Center for Evidence-Based Medicine

Catholic University of Croatia

e-mail: livia.puljak@unicath.hr

phone: + 385 (0) 1 370 66 33
