# Supplementary material for: Perceptions on support, challenges and needs among parents and caregivers of children with developmental disabilities in Croatia, North Macedonia and Serbia: a cross-sectional study
Source: BMC Pediatr. 2024 May 3;24:297. doi: 10.1186/s12887-024-04770-7 (PMC11067112; doi:10.1186/s12887-024-04770-7)
Supplement: Supplementary file 2 — Supplementary Material 2 [file 12887_2024_4770_MOESM2_ESM.docx]

**Supplementary file 2. Study survey in English language**

The survey was administered via SurveyMonkey. This document details which content was showed on each of the 26 pages of the survey, and which questions used skip logic.

**Page 1**

**Erasmus+ project**

*Synergistic education of parents with children with developmental disabilities -SynergyEd*

**Parent and caregiver survey**

The aim of this survey is to investigate the needs of parents/caregivers with children with developmental disabilities and the challenges and difficulties they are facing daily. The results will be used for research and training purposes, in order to provide services for the parents. The survey is one of the activities within the project entitled “Synergistic education of parents with children with developmental disabilities - SynergyEd”, Erasmus+ program. Partners in this projects are Macedonian Association for Applied Psychology from Skopje, Catholic University of Croatia from Zagreb and Institute for Medical Research from Belgrade.

The survey is anonymous.

If you need more information please contact us on the following email address: [contact@simbolikum.mk](mailto:contact@simbolikum.mk)

By clicking on the button “Next”, you will give your consent to participate in the survey.

**Next**

#

# **Page 2**

# **SECTION I: FAMILY DEMOGRAPHIC CHARACTERISTICS**

**1. What is your highest level of education?**

- Primary school
- Secondary school
- University degree
- MSc/PhD

**2. How old are you?**

___________________

**3. If you are married or in partner relationship, what is your spouse’s/partner’s highest level of education?**

- Primary school
- Secondary school
- University degree
- MSc/PhD

**4. How old is your spouse/partner?**

___________________

**5. How many children do you have?**

_____________________

**6. How many children with developmental disabilities do you have?**

______________________

# **Page 3**

# **SECTION II: AFFECTED CHILD CHARACTERISTICS**

*These questions pertain to the nature of the first concerns you had about* your child with developmental disabilities*.* *If you have multiple children with developmental disabilities, answer for the child whose condition you think is the most serious, i.e. who has the greatest difficulties.*

**7. What is your child’s current diagnosis?**

**8. What is your child’s gender?**

- Male
- Female

**9. What is your child’s current age?**

Years:

Months:

## ***First Concerns***

**10. We would like to ask you a little about your child’s early development. How old was your child when you first had a concern about his/her development?**

Years:

Months:

**11. There are many reasons why a parent might be concerned about a child's development. Below is a list of behaviors that can cause a parent to be concerned. Think about the first concerns you had and *select all that apply below*.**

- Had medical problems such as seizures, lack of physical growth, or stomach problems
- Didn’t make eye contact when talking or playing with others
- Didn’t respond when called or didn’t respond to sounds
- Didn’t seem to understand nonverbal communication, such as understanding what you meant by the tone of voice you used or your facial expressions or other body language cues
- Had behavioral difficulties such as sleeping or eating problems, high activity level, wandering, tantrums, aggressive or destructive behavior
- Had problems with coordination or gross motor skills such walking
- Talked later than usual for most children
- Was not talking at all
- Did not talk as well as other children that were the same age
- Some speech skills that he/she had already developed were lost
- Didn't seem to understand what you or other adults said to him/her
- Had problems with fine motor skills such as using scissors or drawing with crayons
- Had difficulty playing or interacting with others
- Had difficulty learning new skills such as toilet training or getting dressed
- Had difficulty learning new things such as the alphabet or numbers
- Other (please specify):

## ***Diagnosis***

**12. How old was your child when he/she was first diagnosed with some of the developmental difficulties?**

Years:

Months:

**13. Who gave your child this initial diagnosis?**

- Primary care physician/Family physician
- Pediatrician
- Pediatric specialist (i.e. developmental pediatrician)
- Psychologist
- Psychiatrist
- Neurologist
- Nurse
- Team of professionals
- Other (Specify: )

**Page 4**

***Pain management***

**14. Does your child experience any physical pain** **related to an underlying condition/problem causing the developmental difficulties, which is long-term (chronic) and lasts for 3 months or longer?**

- Yes (skip logic: go to top of page 5)
- No (skip logic: go to top of page7)
- Don’t know (skip logic: go to top of page7)

**Page 5**

**15. How often does your child feel physical pain?**

- Every day
- Several times a week
- Once a week
- Less than once a week

**16. In which part of the body does the pain occur?**

- Head
- Neck
- Hands
- Body
- Legs
- Multiple body parts

**17. Do you use any medications to resolve this pain?**

Yes (skip logic: go to top of page 6)

No (skip logic: go to top of page 7)

Don’t know (skip logic: go to top of page 7)

**Page 6**

**18. Which pain-relief medicines is your child taking?**

____________________________________________________________________________

**19. Who prescribes those medicines?**

- Specialist (pediatrician, cardiologist, rheumatologist, traumatologist etc.)
- Primary care physician/Family physician
- Somebody else (Please, specify: _____________________________________________)

**Page 7**

***Eating and dietary habits***

##

## **20. How many times does your child usually eat per day?**

## Once

## Twice

## Three times

## Four times

## Five times

## More than five times

##

## **21. Please answer the following according to your child’s particular eating habits?**

|  | Yes | Sometimes | No |
| --- | --- | --- | --- |
| Experiences feelings of hunger during the day |  |  |  |
| Eats a good breakfast |  |  |  |
| Eats meat |  |  |  |
| Eats vegetables |  |  |  |
| Eats fruits |  |  |  |
| Eats dairy products |  |  |  |
| Eats cereals (bread, pasta, rice and baked products) |  |  |  |
| Eats sweets |  |  |  |
| Drinks soft drinks/or sweet beverages |  |  |  |

##

**22. Can you describe your child's appetite?**

- Good
- Fair
- Poor
- Picky

**23. Does your child have mealtime rituals or restrictions (e.g., putting toys or other objects on the table, eating only in the presence of certain conditions, and being very selective about the consistency or color of food and the presentation of food in plate).**

- Yes (skip logic: go to top of page 8)
- No (skip logic: go to top of page 9)

**24. What rituals or restrictions does your child have during meals:**

- Placing toys or other objects on the table
- Eating only under certain conditions
- Very picky about the consistency or color of food
- Great pickiness regarding the presentation of the food on the plate
- Something else ( please, specify what):_______________________________________________

**Page 9**

**25. Do you avoid any food for your child?**

- Yes (skip logic: go to top of page 10)
- No (skip logic: go to top of page 11)

**Page 10**

**26. Which food do you avoid for your child:?**

- Milk
- Eggs
- Meat
- Fruit
- Vegetables
- Something else (please specify what):_________________________________________________

**27. Please specify why you avoid those foods for your child**______________________________________

**Page 11**

**28. Does your child have any food allergies?**

- Yes (skip logic: go to top of page 12)
- No (skip logic: go to top of page 13)

**Page 12**

**29. To what food is your child allergic?**

- Cereals
- Fish
- Eggs
- Milk
- Red fruit
- Peanuts
- Walnuts
- Soy
- Kiwi
- Something else (please specify what): __________________________________________________

**Page 13**

**30. Does your child on some special diet (gluten free, lactose free, intermittent fasting, keto diet etc.)?**

- Yes (skip logic: go to top of page 14)
- No (skip logic: go to top of page 15)

**Page 14**

**31. On what special diet is your child?**

- Gluten-free diet
- Lactose-free diet
- Diet without dairy products
- Diet without eggs
- Hypoallergenic diet
- Vegetarian diet
- Vegan diet
- Rotational diet
- Elementary diet
- Intermittent fasting
- Keto diet
- Macrobiotic nutrition
- Something else (please specify what): _________________________________________________

**32. Who prescribed that special diet**?

- Physician
- Nutricionist
- Nurse
- No one prescribed a diet, we decided alone to use this type of diet
- Someone else recommended this type of diet to you (please specify who): ___________________

**Page 15**

***Supplementation and physical activity***

**33. Does your child use any supplements? (vitamins, minerals, probiotic, melatonin etc.….)?**

- Yes (skip logic: go to top of page 16)
- No (skip logic: go to top of page 17)

**Page 16**

**34. What kind of supplements does the child take?**

- Vitamins
- Minerals
- Probiotics
- Melatonin
- Other (Please specify what):__________________________________________________________

**35. Who prescribed those supplements?**

- Specialist (pediatrician, cardiologist, rheumatologist, traumatologist etc.)
- Primary care physician/Family physician
- No one prescribed supplements, we decided that the child will start taking them
- Someone else, who is not a physician, recommended taking these supplements - please specify who: ______

**Page 17**

**36. Is your child involved in any type of physical activity?**

- Yes (skip logic: go to top of page 18)
- No (skip logic: go to top of page 19)

**Page 18**

**37. If yes, how many hours *per week* is the child involved in physical activity?**

- Less than 1 hour
- 2-3 hours
- 4-5 hours
- 6 hours or more

**Page 19**

**SECTION III: SERVICE ENCOUNTERS**

*These questions pertain to education or other services or treatments that your child may have received in the past or is currently receiving to meet their needs.*

## ***Education services***

**38. What kind of school is your child *currently* enrolled in?**

- Preschool
- Public school
- Private school
- Special school for children with disabilities
- Not enrolled in preschool/school

**39. Does your child receive any additional academic support because of his/her developmental needs?**

- Yes
- No

## ***Other services / assistance***

**40. Does your child currently receive any special assistance from the government/city/municipality etc. because of his/her developmental disabilities?**

- Yes (skip logic: go to top of page 20)
- No (skip logic: go to top of page 21)

**Page 20**

**41. Please indicate what special assistance you receive (you can choose more than one answer)**

- Help from the state
- Help from the city
- Help from the county
- Help from a religious organization
- Some other help (please specify what kind):_____________________________________________

**Page 21**

**42. Do you or any family member currently participate in any family support, advocacy group or organization because of the child’s developmental disabilities?**

- Yes
- No

**43. To what source(s) do you typically turn to get information about your child’s condition? *Select all that apply*.**

- The Internet
- My child’s primary care physician/pediatrician
- My child’s teacher
- Other parents of children with developmental disabilities
- Other providers (i.e. health specialists, therapists) who work with my child)
- Other (please specify):__________________________________________________

**Page 22**

# **SECTION IV: PARENT/CAREGIVER PERCEPTIONS**

##

## ***Access & unmet needs***

*These questions are about all the types of services children may need or use, such as medical care, dental care, specialized therapies, counseling, medical equipment, special education, and early intervention.*

*These services can be obtained in clinics, schools, child care centers, through community programs, at home, and other places.*

**44. During the past 12 months, did you have any difficulties or delays in getting services for your child because the child was not eligible for the services?**

- Yes
- No

**45. During the past 12 months, did you have any difficulties or delays because services the child needed were not available in your area?**

- Yes
- No

**46. During the past 12 months, did you have any difficulties or delays because there were waiting lists, backlogs, or other problems getting appointments?**

- Yes
- No

**47. During the past 12 months, did you have any difficulties or delays because you couldn’t pay for it?**

- Yes
- No

**48. During the past 12 months, did you have any difficulties or delays because you had trouble getting the information you needed?**

- Yes
- No

**49. During the past 12 months, did you have any difficulties or delays for any other reason?**

- Yes (skip logic: go to top of page 23)
- No (skip logic: go to top of page 24)

**Page 23**

**50. Please, could you describe those other reasons?**

__________________________________________________________________________________

**Page 24**

**51. During the past 12 months, how often have you been frustrated in your efforts to get services for your child?**

- Never
- Sometimes
- Usually
- Always

**Page 25**

## ***Family/caregiver impact***

*These questions pertain to the impact that your child’s condition has had on you and other members of your family.*

**52. Has your child’s condition caused financial problems for your family?**

- Yes
- No

**53. Have you or other family members stopped working because of your child’s condition?**

- Yes
- No

**54. Have you or other family members cut down on the hours your work because of your child’s condition?**

- Yes
- No

## ***Stigma***

**55. Please indicate your level of agreement with the following statements**

|  | Strongly disagree | Disagree | I neither agree nor disagree | Agree | Strongly disagree |
| --- | --- | --- | --- | --- | --- |
| **I feel helpless for having a child with developmental disabilities** |  |  |  |  |  |
| **I worry if other people would know I have a child with developmental disabilities.** |  |  |  |  |  |
| **Other people would discriminate against me because I have a child with developmental disabilities.** |  |  |  |  |  |
| **Having a child with developmental disabilities imposes a negative impact on me.** |  |  |  |  |  |

## ***Quality of Life***

**56. Please indicate your level of satisfaction:**

|  | Very dissatisfied | Dissatisfied | Neither dissatisfied nor satisfied | Satisfied | Very satisfied |
| --- | --- | --- | --- | --- | --- |
| **Support for my child by the teachers and school team of experts other than teachers (i.e. psychologist, ocupational therapist, speech therapist to make progress in kindergarden/school** |  |  |  |  |  |
| **Support for my child to make progress at home** |  |  |  |  |  |
| **Support for my child to make friends** |  |  |  |  |  |
| **Relationship with the service providers (speech therapist, psychologists, special rehabilitator and educator) who work with my child** |  |  |  |  |  |
| **Support from your friends** |  |  |  |  |  |
| **Support from your family** |  |  |  |  |  |

## ***Challenges and priorities***

**57. We are now interested in learning what you consider to be the greatest challenges in caring for a child with developmental disabilities. Please select the top 3 challenges from the list below.**

- Challenging behaviors (i.e. self-injury, aggression, tantrums)
- Daily living skills (i.e. toileting, self-feeding, self-care)
- Health problems (i.e. co-occurring physical and/or mental health conditions)
- Sleep problems (i.e. trouble falling asleep, trouble staying asleep)
- Diet/eating/feeding difficulties
- Social interaction difficulties
- Repetitive behaviors/restrictive interests
- Communication difficulties
- Safety concerns (i.e. wandering, climbing)
- Sensory issues (sensitivity on sounds, food textures, touch etc.)
- Other (please specify): ______________________________________________________________

**58. What are the greatest challenges you face in getting support for your child? Please select the top 3 challenges from the list below.**

- Making sure my child receives adequate health care
- Making sure my child receives adequate education
- Making sure my child receives adequate welfare / social supports
- Making sure my child’s basic rights are protected
- Making sure my family and I receive adequate respite
- Other (please specify): ______________________________________________________________

**59. We are also interested in learning what you consider to be the greatest priorities for affected families in your country. Please select the top 3 priorities from the list below.**

- Improved health care services
- Improved education services
- Improved welfare / social services
- Greater rights for individuals with developmental disabilities
- Greater protection of existing rights for individuals with developmental disabilities
- More information about people with developmental disabilities
- Greater in-home support
- Greater community awareness
- Greater opportunities for parent interactions / networking
- Providing assistance to healthy children in the family to better cope with problems related to the children with developmental disabilities
- Provision of psychological help for parents
- A greater number of Institutions/Centers for working with children with developmental disabilities
- More professional staff in existing institutions in the place where the child lives
- Availability of associations for parents of children with developmental disabilities in the place where the child lives
- Better education of existing experts to notice symptoms of developmental delays
- Better education for parents of newly diagnosed children with developmental disabilities in order to teach them how to work with their children
- Other (please specify): ______________________________________________________________

**Page 26**

**Thank you for your participation in this study**
