# Supplementary material for: Perceptions on support, challenges and needs among parents and caregivers of children with developmental disabilities in Croatia, North Macedonia and Serbia: a cross-sectional study
Source: BMC Pediatr. 2024 May 3;24:297. doi: 10.1186/s12887-024-04770-7 (PMC11067112; doi:10.1186/s12887-024-04770-7)
Supplement: Supplementary file 3 — Supplementary Material 3 [file 12887_2024_4770_MOESM3_ESM.docx]

**Supplementary file 3. Description of modifications in the survey compared to the Caregiver Needs Survey**

For this study, we used a survey based on the Caregiver Needs Survey, which was developed by Amy Daniels and National Coordinators of The Southeast European Autism Network (SEAN), as a part of the Global Autism Public Health Initiative of the organization Autism Speaks (<https://www.autismspeaks.org/>). This document describes modifications we made to the Caregiver Needs Survey.

# **1. Demographic characteristics of the family**

Original questionnaire has 3 questions in this section.

## 1.1. Removed items

Za potrebe ove ankete uklonili smo pitanje "Kakav je vaš odnos s djetetom?".

## 1.2. Added items

We added the following 5 items:

**2. How old are you?**

___________________

**3. If you are married or in partner relationship, what is your spouse’s/partner’s highest level of education?**

- Primary school
- Secondary school
- University degree
- MSc/PhD

**4. How old is your spouse/partner?**

___________________

**5. How many children do you have?**

_____________________

**6. How many children with developmental disabilities do you have?**

______________________

We have added these questions to get as precise information as possible and to better understand family dynamics.

# **2. Child characteristics**

In this part, the original questionnaire had 3 parts - general information (current diagnosis, sex and age of the child and his verbal abilities), first fears and diagnosis.

## 2.1. Removed items

In the general information section, the following question "What is your child's verbal ability?" has been removed, since it is not of interest to our project. This section now contains 3 questions.

In the first concerns section, the following question "Who was the first person who was concerned that something was wrong with your child's development?" was removed from the original questionnaire. which was not of interest for our project. This section now contains 2 questions.

In the diagnosis section, the question "How long did you have to travel to get a diagnosis for your child?" was removed. since it is not of interest to our project. This section now contains 2 questions.

## 2.2. Added items:

We have added 3 more sections - pain management, nutrition and eating habits, nutritional supplements and physical activity. These aspects are of great interest for the further activities of the SynergyEd project.

## 2.2.1. Added items about pain

We have added a section related to the presence and treatment of pain with the following questions:

Pain management

14. Does your child experience any physical pain related to an underlying condition/problem causing the developmental difficulties, which is long-term (chronic) and lasts for 3 months or longer?

­ Yes

­ No

­ Don’t know

15. How often does your child feel physical pain?

­ Every day

­ Several times a week

­ Once a week

­ Less than once a week

16. In which part of the body does the pain occur?

­ Head

­ Neck

­ Hands

­ Body

­ Legs

­ Multiple body parts

17. Do you use any medications to resolve this pain?

Yes

No

Don’t know

18. Which pain-relief medicines is your child taking?

____________________________________________________________________________

19. Who prescribes those medicines?

­ Specialist (pediatrician, cardiologist, rheumatologist, traumatologist etc.)

­ Primary care physician/Family physician

­ Somebody else (Please, specify: _____________________________________________)

## 2.2.2. Added items about nutrition

In the nutrition section, we have added the following questions:

Eating and dietary habits

20. How many times does your child usually eat per day?

­ Once

­ Twice

­ Three times

­ Four times

­ Five times

­ More than five times

## 21. Please answer the following according to your child’s particular eating habits?

|  | Yes | Sometimes | No |
| --- | --- | --- | --- |
| Experiences feelings of hunger during the day |  |  |  |
| Eats a good breakfast |  |  |  |
| Eats meat |  |  |  |
| Eats vegetables |  |  |  |
| Eats fruits |  |  |  |
| Eats dairy products |  |  |  |
| Eats cereals (bread, pasta, rice and baked products) |  |  |  |
| Eats sweets |  |  |  |
| Drinks soft drinks/or sweet beverages |  |  |  |

22. Can you describe your child's appetite?

­ Good

­ Fair

­ Poor

­ Picky

23. Does your child have mealtime rituals or restrictions (e.g., putting toys or other objects on the table, eating only in the presence of certain conditions, and being very selective about the consistency or color of food and the presentation of food in plate).

­ Yes

­ No

24. What rituals or restrictions does your child have during meals:

­ Placing toys or other objects on the table

­ Eating only under certain conditions

­ Very picky about the consistency or color of food

­ Great pickiness regarding the presentation of the food on the plate

­ Something else (please, specify what):_______________________________________________

25. Do you avoid any food for your child?

­ Yes

­ No

26. Which food do you avoid for your child:?

­ Milk

­ Eggs

­ Meat

­ Fruit

­ Vegetables

­ Something else (please specify what):_________________________________________________

27. Please specify why you avoid those foods for your child______________________________________

28. Does your child have any food allergies?

­ Yes

­ No

29. To what food is your child allergic?

­ Cereals

­ Fish

­ Eggs

­ Milk

­ Red fruit

­ Peanuts

­ Walnuts

­ Soy

­ Kiwi

­ Something else (please specify what): __________________________________________________

30. Does your child on some special diet (gluten free, lactose free, intermittent fasting, keto diet etc.)?

­ Yes

­ No

31. On what special diet is your child?

­ Gluten-free diet

­ Lactose-free diet

­ Diet without dairy products

­ Diet without eggs

­ Hypoallergenic diet

­ Vegetarian diet

­ Vegan diet

­ Rotational diet

­ Elementary diet

­ Intermittent fasting

­ Keto diet

­ Macrobiotic nutrition

­ Something else (please specify what): _________________________________________________

32. Who prescribed that special diet?

­ Physician

­ Nutricionist

­ Nurse

­ No one prescribed a diet, we decided alone to use this type of diet

­ Someone else recommended this type of diet to you (please specify who): ___________________

## 2.2.2. Added items about physical activity

In the physical activity section, we added the following questions:

36. Is your child involved in any type of physical activity?

- Yes
- No

37. If yes, how many hours *per week* is the child involved in physical activity?

- Less than 1 hour
- 2-3 hours
- 4-5 hours
- 6 hours or more

# **3. Experience with the system**

## 3.1. Removed items

In this section, we have removed the following questions as they are not relevant to our project:

*Health care services*

1. Has your child *ever* received any of the following services or treatments to meet his/her needs? *Select all that apply.*

_____ Behavioral intervention or modification

_____ Sensory integration therapy

_____ Cognitive based therapy

_____ Occupational therapy

_____ Physical therapy

_____ Social skills training

_____ Speech or language therapy

_____ Pharmacotherapy / medication

_____ Other (Specify: ____________________)

_____ Don’t know

2. Is your child *currently* receiving any of the following services or treatments to meet his/her needs? *Select all that apply.*

_____ Behavioral intervention or modification

_____ Sensory integration therapy

_____ Cognitive based therapy

_____ Occupational therapy

_____ Physical therapy

_____ Social skills training

_____ Speech or language therapy

_____ Pharmacotherapy / medication

_____ Other (Specify: ____________________)

_____ Don’t know

3. How many hours of [SELECT FROM SERVICE OR TREATMENT LIST ABOVE] does your child usually receive per week?

4. Do you have to pay for [SELECT FROM SERVICE OR TREATMENT LIST ABOVE]?

_____ Yes, totally

_____ Yes, in part

_____ No

5. Children with learning and developmental conditions work with many different types of service providers to meet their needs. Has your child *ever* worked with the following providers to meet his/her developmental needs? *Select all that apply.*

_____ Audiologist

_____ Behaviorist

_____ Developmental pediatrician

_____ Education specialist

_____ Neurologist

_____ Nutritionist

_____ Psychiatrist

_____ Psychologist

_____ Other (Specify: ____________________)

6. Is your child *currently* working with the following providers to meet his/her developmental needs? *Select all that apply.*

_____ Audiologist

_____ Behaviorist

_____ Developmental pediatrician

_____ Education specialist

_____ Neurologist

_____ Nutritionist

_____ Psychiatrist

_____ Psychologist

_____ Other (Specify: ____________________)

In the **educational service**s section, we have removed the following question "What type of additional academic support is your child currently receiving?" since it is not of interest to our project. This section now contains 2 questions.

In the **other services** section, we have removed the following question, "Do you currently have any education or assistance in managing or addressing your child's needs or conducting your child's treatment?" ” since it is not of interest to our project. This section now contains 4 questions.

## 3.2. Added items

We did not add new items to this part of the questionnaire.

# **4. Impressions of parents/caregivers**

## In this section, we made no changes to the sections on access and unmet needs, impact on caregiver/family, and stigma.

## 4.1. Removed items

In the section on quality of life, we removed the following questions: "For the family to have a good life together, how important is it that my child with autism has support to progress in school?", "For the family to have a good life together, how important is it that my does a child with autism have support to thrive at home?", "For the family to have a good life together, how important is it that my child with autism has support to make friends?", "For the family to have a good life together, how important is it that does my family have a good relationship with the providers who work with my child?” as it is not of interest to this project.

## 4.2. Added items

In order to explore the support that families receive from their environment, we added the following questions:

Please indicate your level of satisfaction with support from your fiends

| Very dissatisfied | Dissatisfied | Neither dissatisfied nor satisfied | Satisfied | Very satisfied |
| --- | --- | --- | --- | --- |

Please indicate your level of satisfaction with support from your family

| Very dissatisfied | Dissatisfied | Neither dissatisfied nor satisfied | Satisfied | Very satisfied |
| --- | --- | --- | --- | --- |

In the **challenges and priorities section**, we have added additional answers to the last question, which reads: We are also interested in learning what you consider to be the greatest priorities for affected families in your country. Please select the top 3 priorities from the list below.

The following responses were added:

- Providing assistance to healthy children in the family to better cope with problems related to the children with developmental disabilities
- Provision of psychological help for parents
- A greater number of Institutions/Centers for working with children with developmental disabilities
- More professional staff in existing institutions in the place where the child lives
- Availability of associations for parents of children with developmental disabilities in the place where the child lives
- Better education of existing experts to notice symptoms of developmental delays
- Better education for parents of newly diagnosed children with developmental disabilities in order to teach them how to work with their children
