# Supplementary material for: Perceptions on support, challenges and needs among parents and caregivers of children with developmental disabilities in Croatia, North Macedonia and Serbia: a cross-sectional study
Source: BMC Pediatr. 2024 May 3;24:297. doi: 10.1186/s12887-024-04770-7 (PMC11067112; doi:10.1186/s12887-024-04770-7)
Supplement: Supplementary file 4 — Supplementary Material 4 [file 12887_2024_4770_MOESM4_ESM.docx]

**Supplementary file 4. Analysis of qualitative content**

Contents

[**Supplementary table 1. Child's current diagnosis** 2](#_Toc153030689)

[**Supplementary table 2. Other concerns about a child's development** 3](#_Toc153030690)

[**Supplementary table 3. Other individuals who determined the child's initial diagnosis** 4](#_Toc153030691)

[**Supplementary table 4. Other sources of special assistance** 5](#_Toc153030692)

[**Supplementary table 5. Other reasons for difficulties or delays** 6](#_Toc153030693)

[**Supplementary table 6. List of questions for which no single answer met the criteria for categorization (i.e. the participants did not provide an answer to the question or the answer was already among those offered, etc.)** 7](#_Toc153030694)

# **Supplementary table 1. Child's current diagnosis**

**(Question 7. What is your child’s current diagnosis?)***

| **Diagnosis** | **Croatia**  **N (%)** | **North Macedonia**  **N (%)** | **Serbia**  **N (%)** | **All**  **N (%)** |
| --- | --- | --- | --- | --- |
| Autism spectrum disorder | 125 (23.02) | 65 (31.70) | 58 (28.15) | 248 (26) |
| Disorders of speech, language and communication | 56 (10.31) | 14 (6.82) | 31 (15.04) | 101 (10.58) |
| Cerebral paralysis and other paralitic/plegic disorders | 30 (5.52) | 38 (18.53) | 19 (9.22) | 87 (9.11) |
| Epilepsy | 32 (5.90) | 19 (9.26) | 14 (6.80) | 65 (6.81) |
| Congenital malformations of the nervous system | 35 (5.90) | 7 (3.41) | 15 (7.28) | 57 (5.66) |
| Hiperkinetic disorders | 44 (8.10) | 5 (2.43) | 3 (1.45) | 52 (5.45) |
| Mixed specific developmental disorders / disharmonious development | 8 (1.54) | 6 (2.92) | 37 (17.96) | 51 (5.34) |
| Delay or absence of the expected stage of development | 20 (3.70) | 16 (7.80) | 4 (1.94) | 40 (4.19) |
| Intellectual disabilities | 26 (4.79) | 6 (2.92) | 7 (3.40) | 39 (4.08) |
| Down syndrome | 23 (4.23) | 8 (3.90) | 2 (0.97) | 33 (3.45) |
| Specific developmental disorders of school skills and motor coordination | 21 (3.86) | 2 (0.97) | 1 (0.48) | 24 (2.51) |
| Disorders of muscles and muscle tone | 17 (3.13) | 1 (0.48) | 3 (1.45) | 21 (2.20) |
| Other specified chromosomal aberrations | 11 (2.02) | 2 (0.97) | 6 (2.91) | 19 (2) |
| Diseases of the urinary system | 12 (2.20) | 4 (1.95) | 0 (0.00) | 16 (1.67) |
| Endocrine diseases | 12 (2.20) | 0 (0.00) | 1 (0.48) | 13 (1.36) |
| Diseases of the musculoskeletal system and connective tissue | 13 (2.39) | 0 (0.00) | 0 (0.00) | 13 (1.36) |
| Diseases of the digestive system | 12 (1.29) | 0 (0.00) | 2 (0.97) | 14 (0.94) |
| Eye diseases | 10 (1.84) | 1 (0.48) | 2 (0.97) | 13 (1.36) |
| Congenital heart defects | 9 (1.10) | 1 (0.48) | 1 (0.48) | 11 (0.83) |
| Ear diseases | 8 (1.47) | 1 (0.48) | 2 (0.97) | 11 (1.15) |
| Metabolic diseases | 6 (1.10) | 1 (0.48) | 0 (0.00) | 7 (0.73) |
| Behavioral disorders | 4 (0.73) | 0 (0.00) | 0 (0.00) | 4 (0.41) |
| Other | 37 (6.81) | 1 (0.48) | 4 (1.94) | 42 (4.40) |

*There are more diagnoses than the number of participants because some participants reported that the child has multiple diagnoses

# **Supplementary table 2. Other concerns about a child's development**

**(Question 11. There are many reasons why a parent might be concerned about a child's development. Below is a list of behaviors that can cause a parent to be concerned. Think about the first concerns you had and select all that apply below. Responses for the answer Other are shown in this Table.)**

| **Response** | **Croatia**  **N (%)** | **North Macedonia**  **N (%)** | **Serbia**  **N (%)** | **All**  **N (%)** |
| --- | --- | --- | --- | --- |
| Mental disorders and other behavioral disorders (sensory problems, lack of concentration, anxiety, waving, fear, misunderstanding, nocturnal urination...) | 17 (3.13) | 12 (5.85) | 15 (7.28) | 44 (4.61) |
| Clear symptoms and clinical signs of congenital malformations and organic or other health problems (Down, CP, spina bifida, vomiting, absence of stool, vision problems, malformations of blood vessels, SHLS, syncope) | 28 (5.15) | 10 (4.87) | 2 (0.97) | 40 (4.19) |
| Tone and motor disorders (hypotonia, tremor, drooling, delay, cessation, sudden development stop) | 14 (2.57) | 11 (5.36) | 10 (4.85) | 35 (3.66) |
| Infections (pneumonia, uroinfection, broncho-obstructions...) | 2 (0.36) | 0 (0.00) | 0 (0.00) | 2 (0.20) |
| Allergies | 1 (0.18) | 0 (0.00) | 0 (0.00) | 1 (0.10) |

# **Supplementary table 3. Other individuals who determined the child's initial diagnosis**

**(Question 13. Who determined the child's initial diagnosis? Responses to the option „Other“)**

| **Response** | **Croatia**  **N (%)** | **North Macedonia**  **N (%)** | **Serbia**  **N (%)** | **All**  **N (%)** |
| --- | --- | --- | --- | --- |
| Speech therapist | 17 (3.13) | 2 (0.97) | 3 (1.45) | 22 (2.30) |
| Educational rehabilitator | 8 (1.47) | 1 (0.48) | 1 (0.48) | 10 (1.04) |
| Physiatrist | 3 (0.55) | 0 (0.00) | 4 (1.94) | 7 (0.73) |
| Gynecologist | 4 (0.73) | 0 (0.00) | 0 (0.00) | 4 (0.41) |
| School medicine doctor | 1 (0.18) | 0 (0.00) | 0 (0.00) | 1 (0.10) |

# **Supplementary table 4. Other sources of special assistance**

**(Question 41. Please indicate what special assistance you receive? Responses for the answer Some other help are shown in this Table.)**

| **Response** | **Croatia**  **N (%)** | **North Macedonia**  **N (%)** | **Serbia**  **N (%)** | **All**  **N (%)** |
| --- | --- | --- | --- | --- |
| Help from other organizations or donations | 0 (0.00) | 1 (0.48) | 2 (0.97) | 3 (0.31) |

# **Supplementary table 5. Other reasons for difficulties or delays**

**(Question 50. During the past 12 months, did you have any difficulties or delays for any other reason? Please, could you describe those other reasons?)**

| **Response** | **Croatia**  **N (%)** | **North Macedonia**  **N (%)** | **Serbia**  **N (%)** | **All**  **N (%)** |
| --- | --- | --- | --- | --- |
| The parent believes that the provision of services is inadequate or discriminatory | 10 (1.84) | 15 (7.31) | 11 (5.33) | 36 (3.77) |
| Other obligations of parents (obligations at work, with another child, etc.) | 4 (0.73) | 1 (0.48) | 5 (2.42) | 10 (1.04) |
| Basic illness of the child (refuses services, delay due to behavior, frequent infections...) | 4 (0.73) | 6 (2.92) | 0 (0.00) | 10 (1.04) |
| Denial of the need for services by family members | 1 (0.18) | 0 (0.00) | 0 (0.00) | 1 (0.10) |

# **Supplementary table 6. List of questions for which no single answer met the criteria for categorization (i.e. the participants did not provide an answer to the question or the answer was already among those offered, etc.)**

| **Question number and the text of the question** |
| --- |
|  |
| **Question 19. Who prescribed those medicines? Responses to the option „Other“)** |
| **Question 24. What rituals or restrictions does your child have during meals (multiple answer were allowed)? Responses to the option „Other“)** |
| **Question 43. To what source(s) do you typically turn to get information about your child’s condition?** **Responses for the answer Other** |
| **Question 57. What you consider to be the greatest challenges in caring for a child with developmental disability? Responses for the answer Other are shown in this Table.** |
| **Question 58. The greatest challenges you face in getting support for your child (three answer were allowed)? Responses for the answer Other are shown in this Table.** |
| **Question 59. What you consider to be the greatest priorities for affected families in your country (three answer were allowed)? Responses for the answer Other are shown in this Table.** |
